# Supplementary figures and images for: An in-silico human cell model reveals the influence of spatial organization on RNA splicing
Source: PLoS Comput Biol. 2020 Mar 25;16(3):e1007717. doi: 10.1371/journal.pcbi.1007717 (PMC7094823; doi:10.1371/journal.pcbi.1007717)

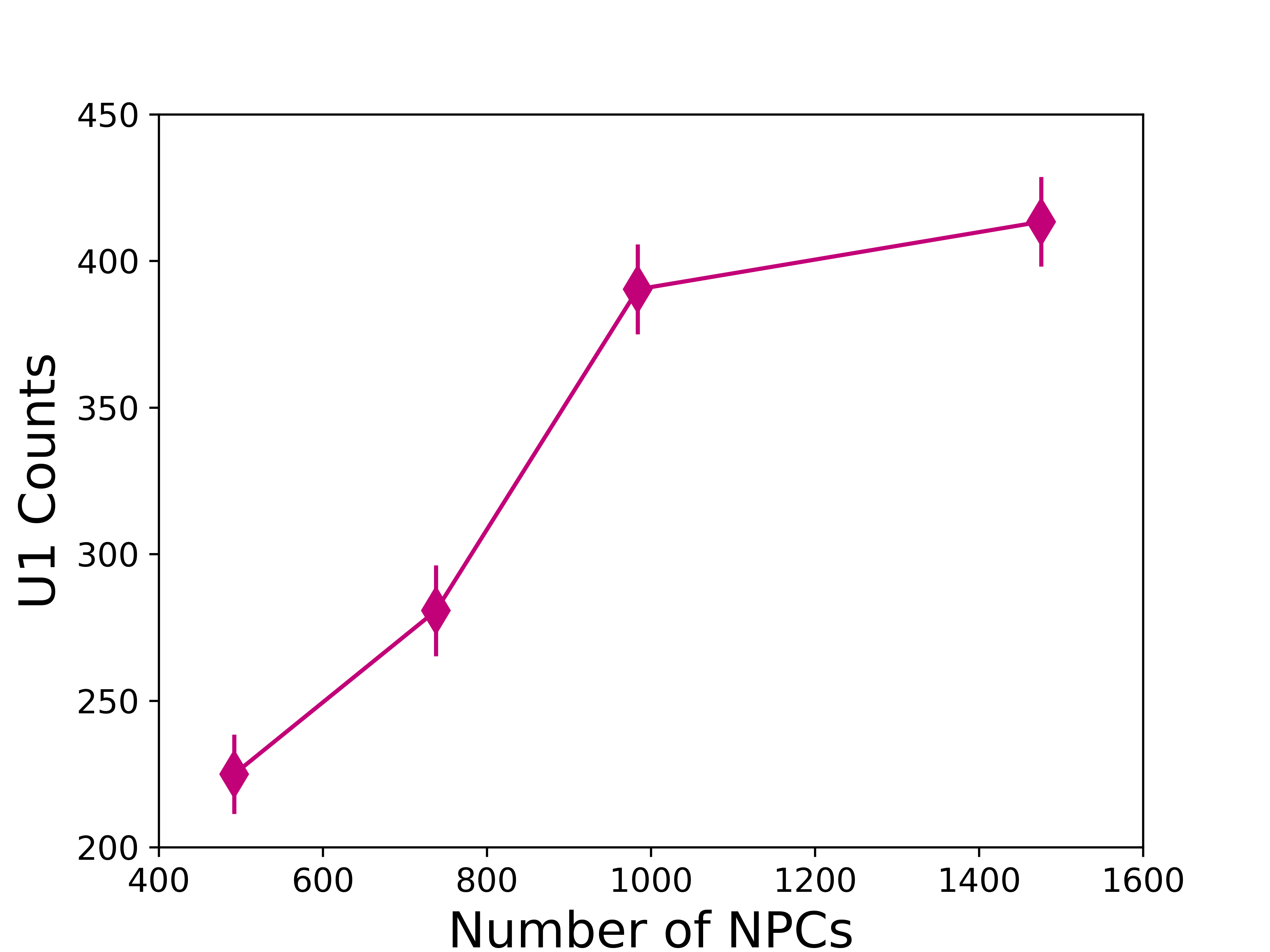

Supplement: S1 Fig — (PNG) [file pcbi.1007717.s003.png]

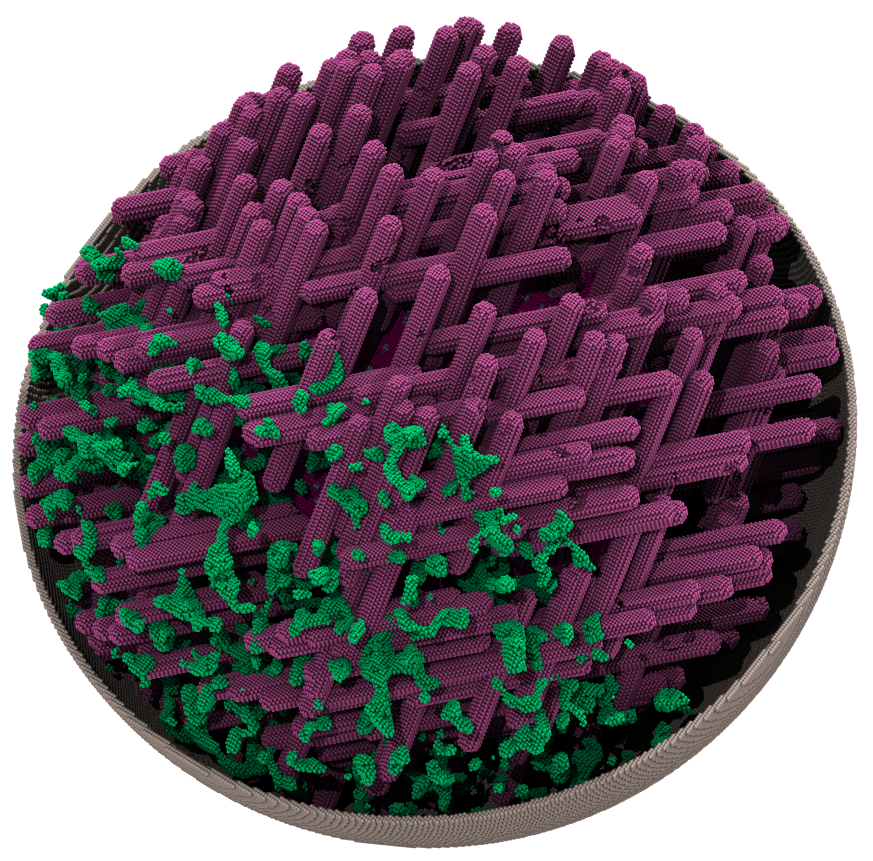

Supplement: S2 Fig — See Methods section for construction details of the mitochondria. (PNG) [file pcbi.1007717.s004.png]

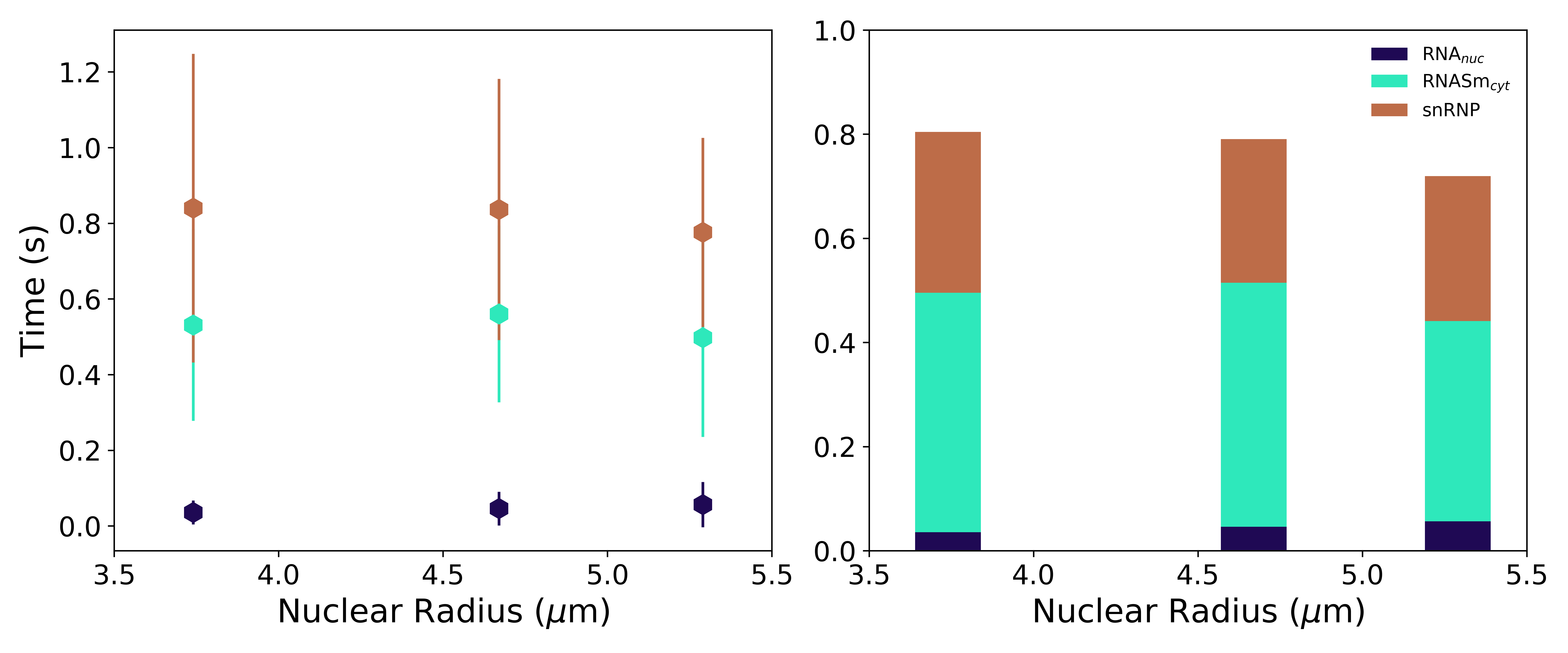

Supplement: S3 Fig — Error bars represent the standard deviations. For each condition, 20 simulation replicates were performed. (PNG) [file pcbi.1007717.s005.png]
