# Supplementary material for: An in-silico human cell model reveals the influence of spatial organization on RNA splicing
Source: PLoS Comput Biol. 2020 Mar 25;16(3):e1007717. doi: 10.1371/journal.pcbi.1007717 (PMC7094823; doi:10.1371/journal.pcbi.1007717)
Supplement: Attachment — Submitted filename: Ghaemi_response_letter.pdf [file pcbi.1007717.s011.pdf]

Dear Drs. Csikász-Nagy and Haugh,

We thank you for considering our manuscript. We also thank the reviewers for their comments that have certainly improved the manuscript. As you will note from our point-by-point response letter, we addressed every reviewers' comments by performing additional work, providing additional information, and rewriting specific sections of our manuscript. The associated revisions in the manuscript are colored in blue, and wherever applicable are also described in the response letter under the heading "Action taken". As a result, our manuscript has now improved significantly.

Editorial review:

We thank the editors for bringing these important points to our attention. Their comments were addressed below.

Specifically, in the revised version please clarify which features of the whole cell model are truly needed to reach the conclusions of the study, since as it seems, most of the claimed data integration into a whole cell model is not used.

Authors response:

We thank the editors for their comments. Our manuscript is intended to serve two purposes: 1. To design and make available a platform for spatial modeling of eukaryotic cells that can utilize experimental data, which can be used or extended by computational biologists/ biophysicists to fit their problems of interest, and 2. To apply the new platform to investigate the spatial effects of one of the most complex eukaryotic processes, RNA splicing, and resolve new aspects of this process through the lens of computational modeling.

As now clearly acknowledged in the manuscript, some of the organelles within the cell are not *actively* involved in the RNA splicing process, namely, mitochondria, Golgi apparatus and endoplasmic reticulum (ER). However, their presence has an important indirect consequence due to excluded volume effects. In fact, the production of splicing particles is reduced by almost a third, because of these indirect effects, which we discuss in the Results section (page 10). Throughout the manuscript and particularly in the caption of Figure 1, we explicitly mentioned the (in)direct involvement of each organelle in our studies.

A further point is that it is stated the software "will be available". We need a more clear statement on this and a specific link where the software can be reached.

Authors response:

The code for setting up the HeLa cell platform is currently available on *GitHub*, together with a user's guide for installing and running the simulations, in addition to modifying the code. The code can be found at: https://github.com/EukaryoticCellBuilder/HeLa_Builder which is publicly available. Thus, researchers should be able to obtain an out of the box model that is easily extensible for their specific projects.

Action Taken:

On pages 19 and 21 the link to the code was mentioned.

Reviewers comments:

Reviewer #1: Whole-cell models that connect molecular biology with phenotype are needed to help researchers integrate disparate data, assemble a comprehensive understanding of biology, and enable experiments that are inaccessible to conventional experimental methods. Toward whole-cell models, Ghaemi *et al.* report a spatially detailed detailed computational model of splicing in HeLa cells and its usage to elucidate several aspects of the role of spatial organization in splicing. The study is innovative for developing one of the first mechanistic models of splicing -- an essential aspect of the biochemical complexity of eukaryotic cells, for reporting some of the longest (15 minutes) spatially detailed simulations of entire eukaryotic cells, and for several novel quantitative analyses of splicing. These innovations are important, especially given the substantial challenges to building and simulating models of whole-cells. The methods are thorough, and the manuscript is well-written.

Authors response:
We thank Dr. Karr and Mr. Shaikh for their enthusiasm for our work. We hope that the code within this manuscript, together with the ideas it conveys, will be helpful for our community. We have improved the manuscript by carefully incorporating their constructive comments, as detailed below.

However, we have several minor comments about several aspects of the manuscript which could be clearer. In addition, we think that the manuscript would be strengthened by an expanded conclusion about the limitations of the reported model and the challenges going forward to building and simulating models that capture more chemical complexity, more intracellular processes, more molecules and spatial compartments, and longer lengths of time.

Authors response:
We thank the reviewers for raising these important issues. Below we provide a list for each of the limitations and challenges that appear in the Conclusions:

Limitations of the current spatially-resolved human cell model
While novel, our model approximates the underlying biophysics, and therefore has some limitations:
1. There is a lack of experimental data for describing some of the rates of individual reactions within the splicing process. To overcome this limitation, we defined approximate lumped reactions (e.g., NPC transport and Sm proteins binding to snRNA[1]) and assigned rates based on either available experimental data[2] or simple models, such as diffusion-limited reactions. A potential alternative to solving this problem is detailed in the next paragraph.

2. Our current model includes nucleus, nuclear speckles and Cajal bodies within it, and cytosolic organelles such as ER, Golgi apparatus, and mitochondria, that are near the nuclear periphery and contribute substantially to the excluded volume effects. However, it does not yet include compartments or structural features such as chromatin, the nucleolus, microtubules, actin filaments, endosomes, peroxisomes, and lysosomes. For the purposes of modeling RNA splicing process, none of these compartments (aside from chromatin) are thought to play a significant role. For a complete spatial model of the human cell, especially when studying processes that are involved with these organelles/compartments, including them will be required.

3. For simplicity we considered a constitutively spliced gene in our studies. To account for complexity within human cells, alternative splicing should be also included which itself requires the inclusion of splice-sites selection. Splice-sites selection is affected by inter- and intracellular signaling pathways.[3]

Challenges of simulating the complete cell cycle of the entire human cell and strategies for overcoming them
There are many challenges to create a dynamical model for the entire human cell. Here, we provide three challenges that in our opinion are the most immediate ones:
1. For simulating the complete life cycle of a eukaryotic cell two additional components will be required: a) inclusion of metabolism, and a more complete description of the genetic information processes (transcription, translation, and DNA replication). Although our code presents a platform for the inclusion of these processes, however, as demonstrated by Karr et al.[4] even for bacterial systems, accounting for these processes will add considerable complexity to the model – especially as they are distributed among different compartments in eukaryotic cells; b) addition of the remaining

organelles such as microtubules, actin filaments and the nucleolus. We envision they can be constructed following a similar approach as outlined in detail in Methods. For representation of sub-compartments such as chromatin, rich sets of experimental data for instance from the 4D nucleome project[5] are becoming available. Such developments will enable modelers to improve the existing model and to capture chromatin dynamics.[6,7] We hope that extension of the model will be a community process and provide other researchers with the tools to make it so (see our comments to the editors).

2. Modeling a comprehensive set of cellular processes requires an extensive amount of computational power, even when performing the simulations on multiple GPUs. Such studies can be accelerated by employing hybrid methodologies. As an example, parts of the cellular processes involving a high concentration of species (e.g., metabolites) can be simulated as ordinary differential equations (ODEs) that can be integrated with stochastic simulations such as chemical master equations (CMEs) for treating the processes like transcription. Recently, a hybrid CME-ODE method was implemented in the LM and can provide a 10-50-fold speedup in comparison with pure CME simulations.[8]

3. For describing an extensive set of biological reactions, especially in eukaryotic cells, the rates of individual reaction steps are often not yet available from experimental data. To overcome this barrier, three approaches can be taken: a) to estimate/infer the rates from computational simulations of each specific reaction using methods such as enhanced sampling techniques[9,10] and quantum mechanical calculations[11]; however, these simulations are computationally expensive and time-consuming; and b) to introduce lumped reactions, based on available experimental data describing the biological processes and time-scale analysis[2]; and c) Bayesian approaches using a multitude of experimental data elements to fit unknown rates.

Action Taken:
We have added the above-mentioned paragraphs to the Conclusions section on pages 17-19 of the revised manuscript.

Minor comments

- Because the reported model represents a portion of a HeLa cell and there are multiple complementary lines of whole-cell modeling research ongoing in the literature, we think would it be helpful for readers to clarify what is meant by a HeLa "whole-cell model" on pp 5.

Authors response:
To address this comment and based on one of the comments of reviewer no. 2, we replaced the term “whole-cell” with “spatially-resolved human cell” in occurrences throughout the manuscript.

At the authors discretion, this could be described in an additional paragraph in the introduction or conclusion. In particular, it would be helpful/interesting to discuss what's missing from the model and what's needed to capture whole cells. For example, what is needed to capture splicing site selection, the combinatorial number of transcripts that could be created from each gene, how to handle the complexity of simulating the entire genome, etc.?

Authors response:
We have addressed this comment by adding two paragraphs to the Conclusions section of the manuscript as discussed above, where we detailed the limitations and challenges in designing a whole-cell model capable of simulating the complete cell cycle of a human cell.
Additionally, the reviewer’s comments stimulated us to explicitly express the assumptions of the spatial model and the reaction schemes in the manuscript. The assumptions include:
1. Our study aims to investigate the RNA splicing process and the main organelles that are *directly* associated with this process including: the nucleus, nuclear speckles, Cajal bodies and parts of the cytoplasm. Therefore, our spatial model of the human cell mainly includes these organelles/ compartments.

2. Because the protein half-life in human cells is ~ 9 hours[12], a quasi-steady cell state is assumed over the 15 minutes of biological time that was simulated. Therefore, processes such as transcription and translation of the genes encoding the proteins involving in the RNA splicing process were not explicitly modeled.
3. We focused on building the groundwork for an adaptable platform for the construction and simulation of spatial models of a human cell. Therefore, the cellular organelles/compartments that have not yet been included in the current version of the model can be readily added through the provided Python code. We anticipate that any required reaction or spatial components will be added by the researchers of our community.

Action Taken:
We added the aforementioned assumptions to the Introduction section on page 4 of the revised manuscript.

- (optional) In the interest of encouraging additional whole-cell modeling research, we think it would be helpful to comment on the extensibility of the model/methods at the end of the conclusions. While the reported model is an advance, realistically it would likely be difficult for most researchers to build upon the implementation, given lack of easy to use whole-cell modeling tools. It would be helpful to comment on how extensible the present model is and what technology is needed to make models like this easier to build and extend.

Authors response:
To build upon our model, we provided documentation within our Python package which describes how to add/ remove reactions; modify the number, size, and morphology of the organelles currently present in the model; and add new organelles. The code can be edited within the widely-used Jupyter notebooks.
As discussed in the Introduction, to perform hour-long simulations of the entire human cell, using GPU acceleration is an inevitable architecture choice. Because our LM software excels on GPU computing, and such technologies are currently available to the majority of researchers, our platform is particularly `primed for extensions to various cell types and longer simulations.

Action Taken:
The above paragraph was added to the Conclusions section under *Model extensibility* subsection on page 19 of the revised manuscript.

- Please clarify several methodological details:
- pp 5: "target volume fractions": Is this intended to mean the modeled volumes of the organelles?

Authors response:
We obtained a fraction of the entire cell volume that is occupied by a specific organelle from the mass spectroscopy data. To clarify, we rewrote this sentence in the manuscript. Additionally, a more detailed description of the approach was presented in the Methods.

Action Taken:
The sentence was rewritten as: "Then for organelles such as the endoplasmic reticulum (ER), we used protein composition percentages of HeLa cell organelles determined by mass spectrometry [13] to determine the fraction of the entire cell volume that is occupied by each organelle (see Methods for details)" on page 5 of the revised manuscript.

- pp 5: "The [essential] components of the cell include: ...": Is this intended to mean essential for the splicing processes that will be investigated?

Authors response:
As mentioned in the model assumptions above, the main organelles that are directly associated with the RNA splicing process including, the nucleus, nuclear speckles, Cajal bodies and parts of the cytoplasm, are present in our model. However, the chromatin and nucleolus were not modelled in this version of the model.

Action Taken:
We rewrote this sentence as: "The essential components of the cell for studying the RNA splicing processes are: the plasma membrane, the cytoplasm, the ER, mitochondria, the Golgi apparatus and the nucleus." on page 5 of the revised manuscript.

- pp 5: "... genes ... were placed around the speckles ...": Please clarify how they were placed.

Authors response:
To clarify this statement, we added the details of gene placement to the manuscript now.

Action Taken:
We rewrote the sentence as: "Actively transcribing genes (black dots in Figure 1-B) were randomly distributed within 0.02 μm of the edge of each nuclear speckle" on page 6 of the revised manuscript.

- pp 7: "... assembles in a [stepwise] manner ..."

Authors response:
To clarify this statement, we added "as described below" to the sentence.

Action Taken:
The sentence was rewritten as: "The spliceosome assembles in a step-wise manner on pre-mRNA transcripts as described below" on page 6 of the revised manuscript.

- pp 7: "... pre-mRNA is [converted] to an mRNA transcript ..."

Authors response:
To avoid confusion, we modified the sentence.

Action Taken:
The sentence was rewritten as: "The splicing reaction occurs (described below) and an mRNA transcript is produced" on page 7 of the revised manuscript.

- pp 8: "... splice site has been chosen ...": Which sites does the model choose?

Authors response:
In our studies we model a constitutive splicing process, namely, all introns are removed by the spliceosome. As we described in the "Co-transcriptional splicing" subsection in page 9, based on ref. [42 of the manuscript] on average each gene (about 28 kbases) has 8 introns. Therefore, subsequently each splice site at the interface of an intron and exon is chosen as the transcriptional machinery moves along the gene (see Figure 2B). To add more details to this statement the sentence was modified.

Action Taken:
The sentence was modified to: "To simplify this network, we assume that a particular splice site has been chosen according to the constitutive splicing process, and focus only on the assembly of the spliceosomal particles on that site and the subsequent splicing reaction." on page 8 of the revised manuscript.

- pp 10: "... production rate of ...": Does this account for the turnover of the particles?

Authors response:
The U1 and U2 spliceosomal particles are known to have half-lives longer than the cell cycle[13,14]. Therefore, in the production rate calculations we did not consider the turnover of the particles. Additionally, U1 (which was the basis of this estimation) is involved in other processes within the cell apart from splicing, hence its abundance is typically higher than other splicing particles.

- Lightening the background in Figure 2A might make the reactions easier to read.

Action taken:
We thank the reviewer for their suggestion. The background Figure 2A has been lightened in the revised manuscript.

- For consistency, should "Complex A" in Figure 2B show one rather than two U1 particles?

Action taken:
We removed the second U1 particle from Figure 2B to avoid confusion.

- The word choice and grammar could be clarified throughout the manuscript. Below are several examples:
- pp 3: "without massively increasing [the] gene count ..."

Action taken:
The reviewers' suggestion was applied to the text.

- pp 3: "The [order of] intronic removal ...". Is this intended to mean temporal order?

Authors response:
In alternative splicing process based on which of the introns are removed, and therefore which of the exons are remaining in the mature mRNA, isoforms of proteins with a variety (and sometimes opposite) functionalities are generated. Because our manuscript does not model the alternative splicing process and to avoid confusion, we simplified the sentence.

Action taken:
The sentence was rewritten as: "A single gene can encode for a variety of functional proteins by a process called alternative splicing." on page 3 of the revised manuscript.

- pp 3: "... coding regions can be shuffled after the removal ...". Do you intend to mean "shuffle" as in exon scrambling? The statement also seems to confuse the order of events; the coding sequence is determined by splicing.

Authors response:
In alternative splicing processes, there are multiple ways by which exons are ligated, such as: exon cassette, mutually exclusive exons, alternative 5' splice site.[15] As mentioned above, because we have not directly modeled the alternative splicing process, we have not explained the details of the complications within this process (*e.g.* the order of the splice site selection, the regulatory processes of the alteration between the splice sites, etc.). To avoid any confusions, we removed this sentence from the revised manuscript.

- pp 3: "... occurs in multiple steps ..., [transport between] .., and terminates ...". We think transport should be plural

Action taken:
The reviewers' suggestion was applied to the text.

- pp 5: The inconsistent use of past and present tense in reference to the distribution of units makes it unclear whether the statements refer to the biology or to the model. Consistent tense would clarify that these statements refer to the model.

Action taken:
The verb tenses were changed.

- pp 7: Correct comma placement in "... reach the cytoplasm where, by a series of complex reactions, they bind ..."

Action taken:

The reviewers’ suggestion was applied to the text.

- pp 8 and others: The first double quote in each pair of quotes should point to the right (e.g., "complex E", "complex B")

Action taken:
The reviewers’ suggestion was applied to the text.

- pp 15: Remove the space in "nano meter" and pluralize

Action taken:
The reviewers’ suggestion was applied to the text.

Reviewer #2: The manuscript by Ghaemi et al describes two main things. First, is a claimed "whole cell" model of a HeLa (mammalian) cell. It would seem to be a whole cell model in the sense that it is spatially resolved, being parameterized in a careful and detailed way from a variety of image based and biochemical composition data. However, the number of biochemical processes considered seems to be quite few to support the claim of a whole cell model (a few RNA splicing reactions).

Authors response:
We thank the reviewer for making this point. As we mentioned above, the main focus of this work is on the formation of splicing components and the core of the RNA splicing process, as it was emphasized in the Abstract and the Introduction. The splicing processes were studied in a spatially resolved cell geometry, but as the reviewer pointed out, not yet truly "whole-cell", because various processes and organelles are still required to be implemented. In the light of the reviewer's comment, we replaced the term "whole-cell" with "spatially-resolved human cell" in occurrences throughout the manuscript. Additionally, in response to similar comment from reviewer # 1 we added the following paragraphs to the Conclusions section to comment on the limitations of our model and the challenges of extending the current model to faithfully represent the "whole cell".

Limitations of the current spatially-resolved human cell model
While novel, our model approximates the underlying biophysics, and therefore has some limitations:
1. There is a lack of experimental data for describing some of the rates of individual reactions within the splicing process. To overcome this limitation, we defined approximate lumped reactions (e.g., NPC transport and Sm proteins binding to snRNA[1]) and assigned rates based on either available experimental data[2] or simple models, such as diffusion-limited reactions. A potential alternative to solving this problem is detailed in the next paragraph.

2. Our current model includes nucleus, nuclear speckles and Cajal bodies within it, and cytosolic organelles such as ER, Golgi apparatus, and mitochondria, that are near the nuclear periphery and contribute substantially to the excluded volume effects. However, it does not yet include compartments or structural features such as chromatin, the nucleolus, microtubules, actin filaments, endosomes, peroxisomes, and lysosomes. For the purposes of modeling RNA splicing process, none of these compartments (aside from chromatin) are thought to play a significant role. For a complete spatial model of the human cell, especially when studying processes that are involved with these organelles/compartments, including them will be required.

3. For simplicity we considered a constitutively spliced gene in our studies. To account for complexity within human cells, alternative splicing should be also included which itself requires the inclusion of splice-sites selection. Splice-sites selection is affected by inter- and intracellular signaling pathways.[3]

Challenges of simulating the complete cell cycle of the entire human cell and strategies for overcoming them
There are many challenges to create a dynamical model for the entire human cell. Here, we provide three challenges that in our opinion are the most immediate ones:
1. For simulating the complete life cycle of a eukaryotic cell two additional components will be required: a) inclusion of metabolism, and a more complete description of the genetic information processes (transcription, translation, and DNA replication). Although our code presents a platform for the inclusion of these processes, however, as demonstrated by Karr et al.[4] even for bacterial systems, accounting for these processes will add considerable complexity to the model – especially as they are distributed among different compartments in eukaryotic cells; b) addition of the remaining organelles such as microtubules, actin filaments and the nucleolus. We envision they can be constructed following a similar approach as outlined in detail in Methods. For representation of sub-compartments such as chromatin, rich sets of experimental data for instance from the 4D nucleome project[5] are becoming available. Such developments will enable modelers to improve the existing model and to capture chromatin dynamics.[6,7] We hope that extension of the model will be a community process and provide other researchers with the tools to make it so (see our comments to the editors).

2. Modeling a comprehensive set of cellular processes requires an extensive amount of computational power, even when performing the simulations on multiple GPUs. Such studies can be accelerated by employing hybrid methodologies. As an example, parts of the cellular processes involving a high concentration of species (e.g.,

metabolites) can be simulated as ordinary differential equations (ODEs) that can be integrated with stochastic simulations such as chemical master equations (CMEs) for treating the processes like transcription. Recently, a hybrid CME-ODE method was implemented in the LM and can provide a 10-50-fold speedup in comparison with pure CME simulations.[8]

3. For describing an extensive set of biological reactions, especially in eukaryotic cells, the rates of individual reaction steps are often not yet available from experimental data. To overcome this barrier, three approaches can be taken: a) to estimate/infer the rates from computational simulations of each specific reaction using methods such as enhanced sampling techniques[9,10] and quantum mechanical calculations[11]; however, these simulations are computationally expensive and time-consuming; and b) to introduce lumped reactions, based on available experimental data describing the biological processes and time-scale analysis[2]; and c) Bayesian approaches using a multitude of experimental data elements to fit unknown rates.

Action Taken:
We have added the above-mentioned paragraphs to the Conclusions section on pages 17-19 of the revised manuscript.

Second, they incorporate in their model a few reactions that describe, mostly in terms of lumped kinetic processes, pre-mRNA splicing. They use spatial kinetic master equation approaches to simulate reaction diffusion mechanisms underlying spliceosome formation, coalescence into nuclear speckles, and production of mature mRNA from pre-mRNA. While splicing is surely a central and important cellular process, the specific questions and rationale for their modeling here was not laid out clearly.

Authors response:
The main points of inquiry of the manuscript are:
1. To investigate the effects of variations in the cellular organelles (specifically nuclear pore complexes and nuclear size) on splicing particles assembly
2. To rationalize the multi-compartmental aspects (cytoplasm and the nucleus) of the assembly of splicing particles
3. To quantitatively characterize the influence of spatial localization of the splicing particles in nuclear speckles and predict how the spatial localization would affect the efficiency of mRNA production and distributions.
4. To predict the effects of active genes distribution around nuclear speckles

Action Taken:
We revised the paragraph in the Introduction section as: "Analogous to the influence of molecular-level heterogeneity, organelle heterogeneity can lead to different cellular phenotypic behaviors. However, the effect of variations in the organelles involved in the spliceosomal particles assembly (e.g., the number of nuclear pore complexes and the size of the nucleus), is yet to be investigated. There is yet another motivation for studies in this realm. It is well-recognized that the particle-assembly in most species, although not yeast, occurs in multiple compartments (the nucleus and cytoplasm) and sub-compartments. What is missing in this context is a quantitative rationale for the shuttling of the precursors of the splicing particles between different compartments. Thirdly, whereas the basic utility of nuclear speckles in pre-mRNA splicing is appreciated, the influence of spatial localization on splicing activity and mRNA production is not quantitatively understood. We anticipate that the localization of splicing components influences the efficiency of splicing" on page 3 of the revised manuscript.

Most of the findings from analyzing the model incorporating these processes seem fairly obvious, such as increasing the number of nuclear pores allows more spliceosomes to be formed because of increased nucleocytoplasmic transport, or that when pre-mRNA are localized with the splicing factors, splicing rates increase significantly.

Authors response:
We thank the reviewer for raising this point. Because this is the first model of its kind ever constructed, and despite the fact that the model is based on a body of experimental data, validation of the model is necessary. Some of our results as the reviewer pointed out are expected, which serves to show that the model is functional. However, we would like to bring attention to a number of non-trivial results such as:

1. we made a quantitative argument for the necessity of the multi-compartmentality of splicing particle formation;
2. we showed that noise (coefficient of variation) in the generated mRNA decreases as the localization percentage of splicing particles in speckles increases;
3. we discussed how the concentration of available splicing particles (or the ratio of splicing particles to pre-mRNA transcripts) affects the observed mRNA production in speckle;
4. from our simulations we suggest a rationale for the observed size and number of nuclear speckles; and
5. we predict that the localization of active gene distribution around the nuclear speckles will likely affect the production of mature mRNA.

We also would like to emphasize that, our manuscript also provides a platform for spatial modeling of eukaryotic cells based on the available experimental data, that can be modified by the community of computational biologists/ biophysicists to study their problems of interest.

Action Taken:
The last paragraph in the Introduction section was modified to: "Our simulations, featuring 15 minutes of biological time of the first spatially-resolved human cell model, explore how cellular organization affects the efficiency of spliceosomal particle formation and pre-mRNA splicing. We find that changes in the number of nuclear pore complexes affect the number of assembled splicing particles, an effect that remains consistent for different nuclear sizes; and quantitatively, the formation of correctly-assembled splicing particles in multiple compartments is more efficient. We also show that even a slight increase in the relative localization of splicing particles in nuclear speckles simultaneously enhances mRNA production and reduces noise in the generated mRNA. We are able to rationalize that the properties of nuclear speckles evolved while subject to physical constraints, such as their size and number. Finally, we predict that the organization of active genes around nuclear speckles affects mRNA production." on page 5 of the revised manuscript.

Moreover, the authors do not seem to use most of the spatial model features incorporated (besides nucleus vs. cytoplasm and nuclear subcompartments such as speckles).

Authors response:
Our manuscript intended to serve two purposes:
1. To design a platform for spatial modeling of eukaryotic cells based on the available experimental data, which can be used or extended by computational biologists/ biophysicists to fit their problems of interest.
2. Utilizing our developed platform, to investigate the spatial effects of one of the most complex eukaryotic processes, i.e. RNA splicing, and magnify new aspects of this process within the lens of computational modeling.

Therefore, despite the fact that some of the organelles within our model are not actively involved in the RNA splicing process, their presence has two consequences: a) in connection to point 1 above, we explicitly show the feasibility of performing the first of its kind model for the geometry of the entire human cell including some biological processes; b) as we discussed in the Results section (page 10) these organelles provide excluded volume effects on the diffusion of reacting species (e.g. G^5, Sm^2, Sm^5, U1(2)snRNA, U1(2)snRNA.G^5, U1(2)snRNA.Sm^5, U1(2)snRNA.Sm^7). The resulting subdiffusive behavior reduces the production of splicing particles by almost one third. The organelles in which there are no reactions are mitochondria, Golgi apparatus and endoplasmic reticulum (ER). Throughout the manuscript and particularly in the caption of Figure 1, we explicitly mentioned the (in)direct involvement of each organelle in our studies.

Action Taken:
We added the following sentence to the caption of Figure 1: "The cytosol, nuclear pore complexes, nuclear speckles and Cajal bodies are directly involved in the RNA splicing processes, whereas ER, mitochondria and Golgi apparatus provide excluded volume effects." on page 6 of the revised manuscript.

Overall, there does not seem to be significant advances or new knowledge presented in the manuscript to warrant publication in its current form.

Authors response:
We hope that by summarizing above the major findings of our manuscript, the reviewer will be convinced that we have put forth new knowledge. We apologize if these points were not made clearer in the original manuscript. In addition to

the aforementioned points, we would like to emphasize on the novel features of our studies that ware identified by Dr. Karr and Mr. Sheikh (reviewers no 1). Specifically, the advances of our work from the current state of the art models can be summarized in the following: 1) The presented model is the first spatial model for the entire human cell ever constructed, which can pave the way for completion and incorporation of other cellular processes by the computational biology community; 2) We showed results of simulations as long as 15 mins of biological time, which is the longest a spatial computational model of a size of a human cell has ever been studied; 3) We studied the effects of spatial organization on the formation of splicing particles and their function. Therefore, we believe that our manuscript provides advancement worth sharing with our community.

-It seems that this model is of a spherical cell, when most HeLa lines are adherent (some are floating).

Authors response:
Trypsin suspends the cells in the buffer which can lead to more spherically-shaped cells[16]. Additionally, in our studies, a key factor impacting our results is the volume exclusion effect of the organelles within the cytoplasm. Therefore, while the shape of the overall cell may play a role, the excluded volume is expected to dominate the effect on the processes we have studied. The role of the cell shape would be a great area of investigation using our new platform, however it is beyond the scope of the current manuscript.

-Mitochondria form large networks, and are usually not thousands of individual compartments (as is currently modeled).

Authors response:
The reviewer is right that mitochondria can form networks during part of the cell cycle. In order to study the effects of the network-like mitochondria on the formation of splicing particles, we constructed a cell that incorporates longer mitochondria resembling a network, while keeping the total volume of the mitochondria constant (see Figure below). We performed two sets of simulations using the new geometry, to study the effects of increase and decrease of nuclear pore complexes (NPCs) on the formation of splicing particles for a nucleus size of 4.67 μm by performing 20 independent simulations.
The results are reported in Table 1 together with the data from the manuscript for the fragmented mitochondria simulations. The new simulation data indicates that despite the network-like geometry for the mitochondria, the increase (decrease) of the number of NPCs leads to the increase (decrease) in the number of splicing particles. Hence, the geometry of the mitochondria does not have a significant effect on the reactions occurring in the cytoplasm. The p-values for the newly obtained data and the data we have presented in the manuscript is reported in the Table 2.

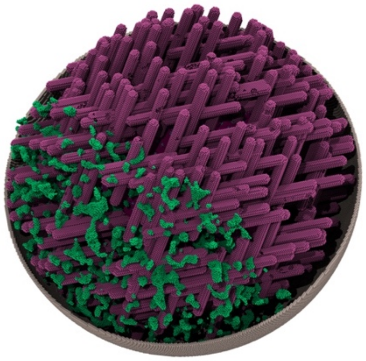

Simulation	Number of U1	Number of U2
+20% NPC in network-like mitochondria	420 ± 21	318 ± 18
+20% NPC in fragmented mitochondria	416 ± 17	319 ± 19
-20% NPC in network-like mitochondria	408 ± 14	292 ± 15
-20% NPC in fragmented mitochondria	397 ± 24	291 ± 11

Table 1: The results of the new sets of simulations with a network-like morphology of the mitochondria as compared to the fragmented-mitochondria that were reported in the manuscript.

Comparing Simulations	p-value
The +20% and -20% NPCs data	0.04
The network-like and fragmented-mitochondria with +20% NPCs	0.5
The network-like and fragmented-mitochondria with -20% NPCs	0.1

Table 2: The p-values of data from various simulation sets.

Action Taken:
The network-like mitochondria structure and associated simulations appeared both in the main text and Supplementary Information. The sentence in the Results section, on page 8 of the revised manuscript, was modified to: "About 2000 rod-shaped mitochondria with dimensions of 0.9 µm × 0.5 µm were randomly placed throughout the cytoplasm, filling ~ 11% of the total volume, additionally, a network-like mitochondria model was also constructed (see Methods section for details)."

In Methods section we added: "A network-like mitochondria was also constructed by randomly placing 2.95 µm-long rods that can cross each other, while keeping the total volume of the mitochondria constant, as shown in Figure S2." on page 20 of the revised manuscript.

A subsection with the figure and tables presented above was added to the Supplementary Information.

-Liquid liquid phase separation is known to be caused by intrinsically disordered proteins and/or multi-valent interactions, but no such mechanisms were used to invoke nuclear speckles, which were claimed to be separate liquid phases.

Authors response:
The focus of our model is not on the mechanisms of nuclear speckles formation *per se*, but rather on the localization effects within these regions on the reactions and noise of the products, i.e., mature RNA molecules. We made no claims on the phase of the nuclear speckles and we added a sentence to the Results to explicitly mention this point. As the reviewer is aware, the formation of nuclear speckles is an exciting topic of ongoing research, and the components are being mapped out by techniques such as mass spectroscopy. This may allow addition of nuclear speckle dynamics to future versions of the model, and we are on the lookout for such data.

References:

(1) So, B. R.; Wan, L.; Zhang, Z.; Li, P.; Babiash, E.; Duan, J.; Younis, I.; Dreyfuss, G. A U1 snRNP–specific assembly pathway reveals the SMN complex as a versatile hub for RNP exchange. Nat. Struct. Mol. Biol. 2016, 23 (3), 225–230.
(2) Alpert, T.; Herzel, L.; Neugebauer, K. M. Perfect timing: splicing and transcription rates in living cells. Wiley Interdiscip. Rev. RNA 2017, 8 (2), 1–12.
(3) Fu, X.-D.; Ares, M. Context-dependent control of alternative splicing by RNA-binding proteins. Nat. Rev. Genet. 2014, 15 (10), 689–701.
(4) Karr, J. R.; Sanghvi, J. C.; Macklin, D. N.; Gutschow, M. V; Jacobs, J. M.; Bolival, B.; Assad-Garcia, N.; Glass, J. I.; Covert, M. W. A whole-cell computational model predicts phenotype from genotype. Cell 2012, 150 (2), 389–401.
(5) Dekker, J.; Belmont, A. S.; Guttman, M.; Leshyk, V. O.; Lis, J. T.; Lomvardas, S.; Mirny, L. A.; O'Shea, C. C.; Park, P. J.; Ren, B.; et al. The 4D nucleome project. Nature 2017, 549 (7671), 219–226.
(6) Rosa, A.; Di Stefano, M.; Micheletti, C. Topological Constraints in Eukaryotic Genomes and How They Can Be Exploited to Improve Spatial Models of Chromosomes . Frontiers in Molecular Biosciences . 2019, p 127.
(7) Lin, D.; Bonora, G.; Yardımcı, G. G. Computational methods for analyzing and modeling genome structure

and organization. WIREs Syst. Biol. Med. 2019, 11 (1), e1435.
(8) Bianchi, D.; Peterson, J.; Earnest, T.; Hallock, M.; Luthey-Schulten, Z. A Hybrid {CME}-{ODE} Method for Efficient Simulation of the Galactose Switch in Yeast. {IET} Syst. Biol. 2018.
(9) Laio, A.; Parrinello, M. Escaping free-energy minima. Proc. Natl. Acad. Sci. U. S. A. 2002, 99 (20), 12562–12566.
(10) Kästner, J. Umbrella sampling. WIREs Comput. Mol. Sci. 2011, 1 (6), 932–942.
(11) Miller, W. H. Beyond transition-state theory: a rigorous quantum theory of chemical reaction rates. Acc. Chem. Res. 1993, 26 (4), 174–181.
(12) Eden, E.; Geva-Zatorsky, N.; Issaeva, I.; Cohen, A.; Dekel, E.; Danon, T.; Cohen, L.; Mayo, A.; Alon, U. Proteome Half-Life Dynamics in Living Human Cells. Science (80-.). 2011, 331 (6018), 764 LP – 768.
(13) Sauterer, R. A.; Feeney, R. J.; Zieve, G. W. Cytoplasmic assembly of snRNP particles from stored proteins and newly transcribed snRNA's in L929 mouse fibroblasts. Exp. Cell Res. 1988, 176 (2), 344–359.
(14) Berg, M. G.; Singh, L. N.; Younis, I.; Liu, Q.; Pinto, A. M.; Kaida, D.; Zhang, Z.; Cho, S.; Sherrill-Mix, S.; Wan, L.; et al. U1 snRNP Determines mRNA Length and Regulates Isoform Expression. Cell 2012, 150 (1), 53–64.
(15) Vuong, C. K.; Black, D. L.; Zheng, S. The neurogenetics of alternative splicing. Nat. Rev. Neurosci. 2016, 17 (5), 265–281.
(16) Fujioka, A.; Terai, K.; Itoh, R. E.; Aoki, K.; Nakamura, T.; Kuroda, S.; Nishida, E.; Matsuda, M. Dynamics of the {R}as/{ERK} {MAPK} Cascade as Monitored by Fluorescent Probes. J. Biol. Chem. 2006, 281 (13), 8917–8926.
