# Supplementary material for: An in-silico human cell model reveals the influence of spatial organization on RNA splicing
Source: PLoS Comput Biol. 2020 Mar 25;16(3):e1007717. doi: 10.1371/journal.pcbi.1007717 (PMC7094823; doi:10.1371/journal.pcbi.1007717)
Supplement: Attachment — Submitted filename: Ghaemi_Response2.pdf [file pcbi.1007717.s012.pdf]

Dear Drs. Csikász-Nagy and Haugh,

We thank you again for considering our manuscript. We also thank the reviewers for their further comments that have improved our manuscript. Below, we provide a point-by-point response to reviewers' comments. The associated revisions in the manuscript are colored in blue. We hope that the additions/modifications we have made to the manuscript and the associated code will satisfy the reviewers.

Reviewers comments:

Reviewer #1: The authors have addressed our major concerns. However, we feel that the manuscript would be strengthened by clarifying the purpose and goals of the study in the abstract, author summary, and introduction.

Authors response: The purpose and goals of this study are highlighted in the Abstract: "Our goal is to model the effect of spatial organization of membrane-less organelles (specifically nuclear speckles) and of organelle heterogeneity on splicing particle biogenesis in mammalian cells."

And in the Introduction section: "Analogous to the influence of molecular-level heterogeneity, organelle heterogeneity can lead to different cellular phenotypic behaviors. However, the effect of variations in the organelles involved in the spliceosomal particles assembly (e.g., the number of nuclear pore complexes and the size of the nucleus), is yet to be investigated. There is yet another motivation for studies in this realm. It is well-recognized that the particle-assembly in most species, although not yeast, occurs in multiple compartments (the nucleus and cytoplasm) and sub-compartments. What is missing in this context is a quantitative rationale for the shuttling of the precursors of the splicing particles between different compartments. Thirdly, whereas the basic utility of nuclear speckles in pre-mRNA splicing is appreciated, the influence of spatial localization on splicing activity and mRNA production is not quantitatively understood. We anticipate that the localization of splicing components influences the efficiency of splicing."

In addition, although the HelaBuilder tutorial is easy to follow and the model is extensible *in principle*, in practice the procedural definition of the model and the lack of annotation within the model code will make it challenging for others to expand upon the model.

Authors response: We have added extensive annotations throughout the code and have incorporated two extra examples in the documentation that show how to adapt our platform to other scenarios. The first example demonstrates how an alternate reaction system can be modelled within the HeLa cell, and the second, details how a yeast cell can be generated with our platform. Additionally, we have also added a Github webpage and a contact email address on the webpage to answer researchers' questions as they use the model. The webpage can be accessed at: https://eukaryoticcellbuilder.github.io/HeLa_Builder/. Our efforts in completing the cell builder platform will continue in the future in the hope that this tool can be useful for our community.

In the interest of transparency, we think its important to acknowledge that much more needs to be done to make this model and other really extensible.

Authors response: To acknowledge the reviewer comment, we have added the following paragraph to the Conclusions section of the manuscript.

"Having said that, our platform presents one of the many steps required to be able to seamlessly integrate various models and fully simulate the complexity of a eukaryotic cell across different length and time scales. Such an integration can finally lead to the prediction of inter- and intra-cellular emergent phenomena."

Minor comments
- knwon is mispelled on page 3

Authors response: The reviewers' suggestion was applied to the text.

Reviewer #2: The authors have substantially revised the manuscript for clarity which has much improved it. The specific application to splicing remains somewhat lacking in this reviewer's opinion (mainly in terms of new biological knowledge). However, the bigger picture aspects of the manuscript, such as a spatially resolved mammalian cell model scaffold with available code for extension, and pushing the boundaries of simulation time for such a model, are appreciated and should benefit the literature and community.

Authors response: We thank the reviewer for her/his comments which have substantially improved our manuscript.
